# The role of jasmonates in plant root growth and development

**DOI:** 10.3389/fpls.2026.1836825

**Published:** 2026-05-13

**Authors:** Wei Sun, Bin Wu, Chunxue Kong, Guiqing Xiao, Rongfeng Huang, Hua Qin

**Affiliations:** 1 Shandong Provincial Key Laboratory of Plant Stress, College of Life Sciences, Shandong Normal University, Jinan, China; 2 College of Bioscience and Biotechnology, Hunan Agricultural University, Changsha, China; 3 Biotechnology Research Institute, Chinese Academy of Agricultural Sciences, Beijing, China

**Keywords:** crosstalk, jasmonates, phytohormone, regulatory network, root growth, signaling transduction

## Abstract

Root growth is correlated to plants fitness and productivity, and adaption to stressful environments. Its growth is a complex and precisely regulated process that involves the interplay of multiple phytohormones. Jasmonates (JAs), as key defense hormones, not only participate in the response of plants to biotic and abiotic stresses, but also play a critical role in root growth and development. This review provides insights into the progress being made in understanding the role of JA in root growth and development, highlighting the integration of JA with other phytohormones, including auxin, ethylene, cytokinin, abscisic acid, gibberellin, and brassinosteroid. These hormone signaling pathways form a complicated regulatory network to shape the root system structure. Understanding the regulatory network of JA-regulated root growth will be instrumental for elucidating the molecular mechanism underlying plant growth-defense tradeoff, which will accelerate future crop breeding programs to obtain cultivars that combine robust defenses while maintaining normal yields.

## Highlights

Jasmonates exert its regulatory roles on root growth by integrating with auxin, ethylene, cytokinin, abscisic acid, gibberellin, and brassinosteroid biosynthesis or signaling pathways through its core signaling module COI1-JAZ-MYC.

## 1 Introduction

As an underground organ of plants, roots anchor the plant to its growth substrate, absorb water and nutrients from the soil, and sense and respond to changing environmental situations. Plants with robust root system often exhibit obvious growth advantages and high resistance to abiotic stresses, thereby reducing seedling mortality and increasing yield (Gao et al., 2023; Uga et al., 2013). There are two main types of root systems in plants: taproot systems and fibrous root systems. In dicotyledonous plants such as *Arabidopsis thaliana*, a taproot system develops through a hierarchical pattern of primary and lateral root growth. By contrast, monocot crops such as *Oryza sativa* L. undergo an early developmental transition in which the embryonic primary root degenerates and is replenished by a fibrous root system predominantly consisting of post-embryonic adventitious roots, which are also known as crown roots in cereals (Coudert et al., 2010; Qin et al., 2022). With the deepening of the research on roots, many scientists are starting to see roots as central to their efforts to produce crops with a better yield. Moreover, it is becoming increasingly evident that optimization of root architecture for resource capture is vital for enabling the next green revolution (Gewin, 2010).

During the life cycle of plants, root systems are continuously renewed by the initiation and elongation of new roots, thus ensuring efficient acquisition of water and nutrients from the heterogeneous soil environment and facilitating the plant’s adaptation to biotic and abiotic stresses, which contributes immensely to plant fitness and sustainable crop production under changing soil environment (Karlova et al., 2021; Voothuluru et al., 2024). Continuous root growth is sustained by cell division in the root apical meristem and cell elongation of cells that leave the root meristem (Perini et al., 2012; Petricka et al., 2012), this process is influenced by internal developmental signals and external environmental factors, and phytohormones are central regulators in this process (Li et al., 2025; Liu and Fu, 2024; Qin et al., 2019).

Jasmonates (JAs), including the lipid-derived hormone jasmonoyl-L-isoleucine (JA-Ile) and its metabolic precursors and derivatives, were primarily recognized as “defense hormones” in wounding and defense responses. Accumulating studies shows that JAs also play a crucial role in root growth and development (Gasperini et al., 2015; Li et al., 2022). Studies have shown that JA exhibits dual regulatory characteristics of “inhibition and promotion” during root growth and development, which is achieved through the interaction with other phytohormones such as auxin, ethylene, brassinosteroid, etc (Han et al., 2023; Huang et al., 2010; Li et al., 2022; Qin et al., 2019). In this review, we summarize the current research progresses concerning the regulatory effects of JA and its crosstalk with other phytohormones during root growth and development, which will facilitate a better understanding of the function of JA in root development and provide guidance for genetic improvement of crop root systems to enhance crop yield and adaptation to stressful environmental conditions.

## 2 Biosynthesis and signal transduction of JA

### 2.1 Biosynthesis of JA

**Figure 1:**
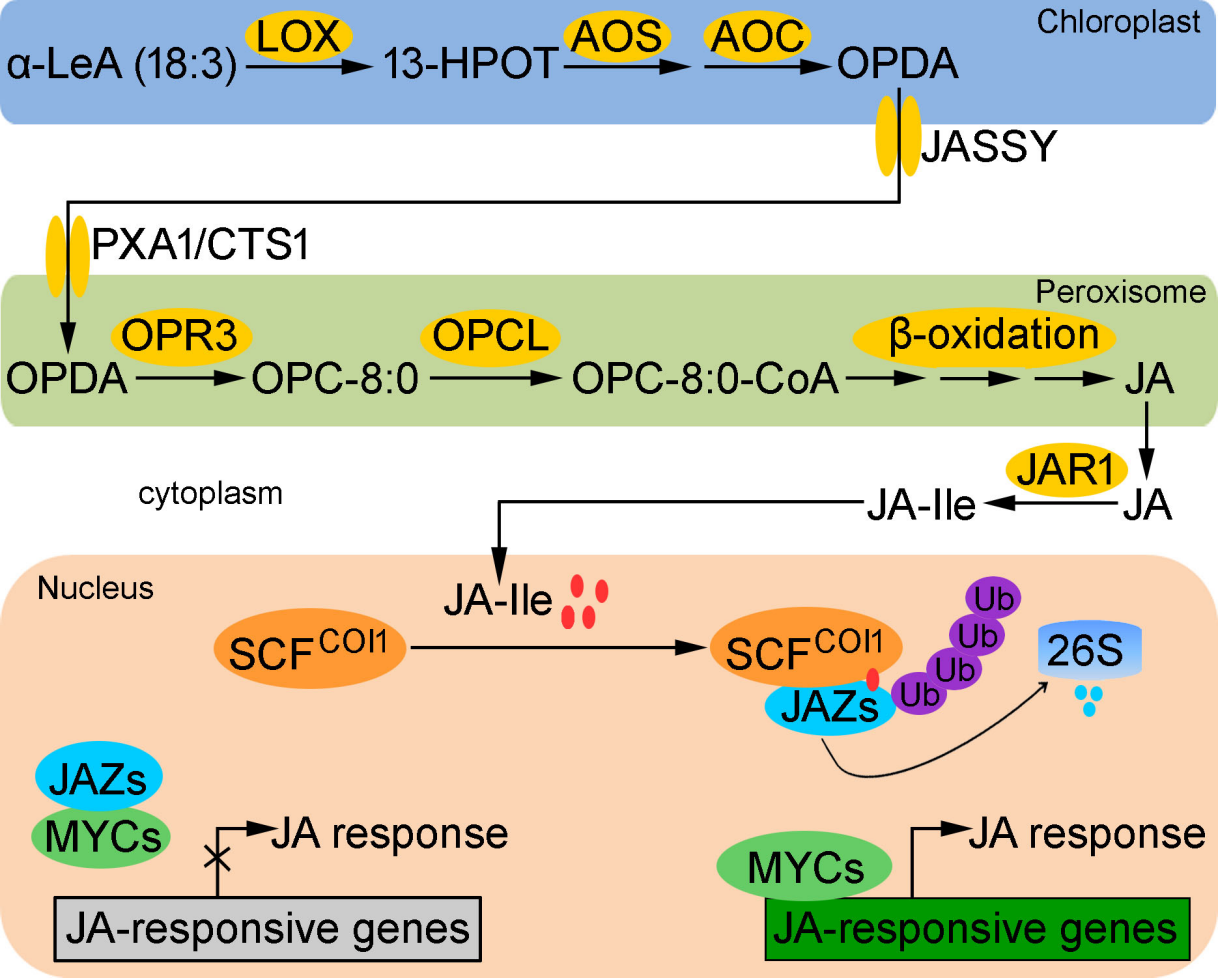
Schematic diagram of jasmonate (JA) biosynthesis and signaling pathway. The biosynthesis of JA occurs sequentially in chloroplast and peroxisome from α-linolenic acid (α-LeA/18:3) under the catalysis of JA biosynthetic enzymes. Peroxisomal JA is then transported to the cytoplasm, where diverse JA derivatives are produced, including the bioactive JA-Ile. JA-Ile then enters the nucleus to induce the interaction of CORONATINE INSENSITIVE 1 (COI1) with JASMONATE ZIM-DOMAIN (JAZ) proteins, leading to the ubiquitination and degradation of JAZ proteins through the 26S proteasome pathway, thereby releasing MYCs to activate the expression of JA-responsive genes and JA responses. Ub, ubiquitin.

The biosynthesis of JA occurs in chloroplasts and peroxisome, initiating with the conversion of α-linolenic acid (α-LeA/18:3) into (13S)-hydroperoxyoctadecatrienoic acid (13-HPOT) by 13-lipoxygenase (LOX), which is then converted into 12-oxo-phytodienoic acid (OPDA) by allene oxide synthase (AOS) and allene oxide cyclase (AOC). OPDA is transported from chloroplasts to the peroxisomes by the chloroplast envelope-localized transporter JASSY (Guan et al., 2019), peroxisomal ABC-transporter 1 (PXA1), and COMATOSE1 (CTS1) (Theodoulou et al., 2005). In the peroxisome, OPDA is catalyzed by OPDA reductase 3 (OPR3) to yield 3-oxo-2-(cis-2’-pentenyl)-cyclopentane-1-octanoic acid (OPC-8:0). OPC-8:0 is then activated to OPC-8:0 CoA by OPC-8:0 CoA ligase (OPCL), and subsequently JA is produced by three cycles of β-oxidation and exported to the cytoplasm (Sheard et al., 2010). In the cytoplasm, JA undergoes further modifications to form various derivatives, the most significant of which is jasmonoyl-L-isoleucine (JA-Ile). JA-Ile is recognized as the most biologically active form of JA, exerting physiological functions in plants by binding to receptors to activate downstream signals (Wasternack and Strnad, 2016) (**Figure 1**).

### 2.2 Signal transduction of JA

The core JA signaling pathway involves the F-box protein CORONATINE INSENSITIVE 1 (COI1) and the JASMONATE ZIM-DOMAIN (JAZ) proteins (Sheard et al., 2010). Generally, JA-Ile remains at a low level, leading to the accumulation of JAZ proteins, which physically bind to MYC transcription factors to repress their activity through two distinct mechanisms (Chini et al., 2007; Guan et al., 2019; Yan et al., 2007) (**Figure 1**). Briefly, MYC-bound JAZ proteins recruit the co-repressors TOPLESS (TPL) and TPL-Related (TPR) proteins, either directly through ETHYLENE-RESPONSE FACTOR-associated amphiphilic repression (EAR) motifs located at the N-terminal region of the JAZ proteins, or indirectly via an interaction with the adaptor protein NOVEL INTERACTOR OF JAZ (NINJA) (Pauwels et al., 2010; Shyu et al., 2012). TPL in turn recruits histone deacetylases and histone methyltransferases to silence gene expression, thereby obstructing JA signaling transduction (Wang et al., 2013). Upon stimulation, JA-Ile rapidly accumulated, which promotes the binding of JAZ proteins to the COI1 component of the Skp1/Cul1/F-box (SCF) E3 ubiquitin ligase complex (SCF^COI1^), resulting in the ubiquitylation and degradation of JAZ proteins (Chini et al., 2007). JAZ degradation unmasks the MED25 binding site on MYC, allowing the formation of the transcription preinitiation complex with RNA polymerase II and thereby activating core JA signaling (Çevik et al., 2012; Chen et al., 2012). Moreover, MYC undergoes pronounced conformational changes when bound to the conserved Jas motif of the JAZs repressors, thereby preventing the interaction between MYC and the MED25 subunit of the transcriptional Mediator complex (Zhang et al., 2015a), suggesting a dynamic molecular switch mechanism that governs the repression and activation of JA signaling pathway.

## 3 The diversity role of JA in root growth

JA is a well-known defense hormone, and it also plays an important role in plant root development. Studies have shown that JA has biphasic effects on root growth; namely, inhibiting primary root elongation, whereas promoting adventitious roots, lateral roots, and root hairs development (Han et al., 2023; To et al., 2022).

### 3.1 The role of JA in primary root growth

The primary root, initiated during embryo development, is fundamental for seedling establishment as it provides the seedling with its sole source of anchorage and water/nutrient absorption. In dicot plants such as *Arabidopsis thaliana*, the continuous growth of the primary root is required for plants to complete their lifecycles, whereas in monocot plants such as rice, the primary root grows rapidly for 7–10 days after germination and then dies when crown roots take over. Accumulating studies showed that JA inhibits primary root growth, and this effect is primarily mediated by the JA-COI1-JAZ-MYC2 signaling module (Han et al., 2023; Huang et al., 2017). Mutations in COI1 and MYC2, or overexpression of *JAZs* results in reduced sensitive to JA-induced primary root growth inhibition. Conversely, overexpression of *MYC2* or mutations of JAZs leads to increased JA-induced root growth inhibition (Gasperini et al., 2015; Guo et al., 2018; Thireault et al., 2015; Xu et al., 2002), suggesting the negative role of JA in primary root elongation. In rice, *osmyc2* mutants exhibit longer primary root and reduced sensitivity to JA (Zu et al., 2024), whereas transgenic rice plants overexpressing *OsMYC2* exhibited a dwarf phenotype and were more resistant to bacterial blight (Uji et al., 2016), suggesting the important role of JA in coordinating plant growth and defense responses.

Root growth is largely dependent on the rate of cell proliferation in the root apical meristem (Chen et al., 2011; Zhang et al., 2015b). The quiescent center (QC), located in the center of the root tip, is consist of several mitotically inactive cells and function as organizer of the root stem cell niche. Its quiescence is fundamental to maintain root structure and meristem function (Heyman et al., 2013). JA treatment promotes the division of QC cells, thereby inhibiting cell proliferation in root meristem to inhibit primary root elongation, this effect of JA is executed by direct repressing of MYC2 to the *PLETHORA1* (*PLT1*) and *PLT2* genes, which encode the AP2 class of transcription factors that are essential for QC specification and stem cell activity (Chen et al., 2011). Another AP2/ERF family transcription factor ERF115, which has been reported to control root QC cell division, was directly induced by JA in a COl1- and MYC2-dependent manner. *erf115* mutant showed reduced JA-mediated QC divisions (Heyman et al., 2013; Zhou et al., 2019). All these studies suggest that JA inhibits primary root elongation by inducing the division of QC cells to reduce root meristem activity, and basal level of JA and intact JA signaling is crucial for QC quiescent and primary root elongation.

### 3.2 The function of JA in adventitious roots development

Adventitious roots (ARs) are the main components of the fibrous root system, which are grown from differentiated cells of non-root organs, such as stems, hypocotyls, or leaves. It formed either during the intrinsic development or in response to environmental stresses (Steffens and Rasmussen, 2016). ARs emerge from the stem nodes of cereals are called crown roots (CRs) (Coudert et al., 2010). These roots can be continuously renewed throughout the plant’s life and profoundly impact root system architecture and subsequent aboveground biomass accumulation, thus it has become a prime target for genetic improvement of crop root systems.

Numerous studies have shown that JA is involved in regulating AR development (Dob et al., 2021; To et al., 2022). In *Arabidopsis thaliana*, genetic analysis has evidenced that the COI1-dependent MYC2-mediated JA signaling inhibited the intact hypocotyl-derived AR initiation. Moreover, JA represses AR initiation by activating the expression of *ERF115* and its closest homologs *ERF113* and *ERF114*. The *ERF115* overexpressing lines developed extremely few ARs, whereas repressing *ERF115* expression promoted ARs development (Lakehal et al., 2020). Contrary to its function in *Arabidopsis thaliana*, exogenous JA treatment increased CR number in rice, and the germin-like protein OsGER4 is required for CR development under exogenous JA treatment. *osger4* mutants produce significantly fewer CRs and fewer primordia under long treatment with JA (To et al., 2022). In wounded plants, JA has been demonstrated to act as a master trigger for promoting AR formation (Liu et al., 2025). The formation of ARs from stem cuttings or leaf cuttings is a key step in vegetative (or clonal) propagation, which is widely used in forestry, agriculture, and horticulture to propagate elite germplasm relatively quickly and cheaply. Collectively, these investigations show that JA has diverse roles in AR development, depending on developmental context and plant species, highlighting its potential as a target for genetic manipulation to improve root traits.

### 3.3 The role of JA in lateral roots and root hairs development

Lateral roots (LRs) development is initiated by the asymmetric division of the pericycle cells, and subsequent divisions result in the formation of LR primordial (LRP). Ultimately, the LRP break through the epidermal cells to become new LRs (Yu et al., 2016). Root hairs (RHs) are unicellular extensions of root epidermal cells (Dolan, 2017). LRs and RHs are the important root components, as they enlarge the soil-root interface, thus increasing water and nutrient uptake, and improving soil anchorage. The development of LRs and RHs is precisely regulated by intrinsic signals and environmental cues, and in particular by diverse endogenous hormones (Cai et al., 2014; Sui et al., 2025; Sun et al., 2009).

JA significantly promotes the formation of LRs and RHs, thereby increasing the surface area of the root system and enhancing the plant’s capacity to absorb water and nutrients (Cai et al., 2014; Zhu et al., 2006). In *Arabidopsis thaliana*, JAZs interacted with ROOT HAIR DEFECTIVE 6 (RHD6) and RHD6 LIKE1 (RSL1), two transcription factors that are essential for RH development, to repress the transcriptional function of RHD6 and interfere with the interaction of RHD6 with RSL1 to repress RH development. Accordingly, disruption of JAZ repressors promotes RH development. JA-induced root hair development was severely disrupted in rhd6 rsl1 mutants, and overexpression of *RHD6* largely rescued the root hair defects of JAZ-accumulating plants (Han et al., 2020). Moreover, ERF114/115/109 regulates JA signaling through interacting with JAZ8, disrupting the formation of the MYC2/3/4-JAZ8 and RHD6-JAZ8 complex to regulate JA-promoted LR formation and RH growth (Cai et al., 2014; Sui et al., 2025). In rice, exogenous application of 2 µM JA increased the LR density by 1.6 fold (Wang et al., 2002). Deficiencies of macronutrients (N, P and K^+^) enhance bioactive JA accumulation in rice root to promote LRs and RHs development to enhance the acquisition of nutrients from the soil, thereby alleviating the effects of nutrient deficiency on plant growth and crop yield (Deepika and Singh, 2021; Kamali et al., 2025; Singh et al., 2020), suggesting that precisely manipulation of JA to improve crop root system could reduce fertilizer input without yield penalties, thus achieving green and efficient cultivation goals.

### 3.4 The role of JA in regeneration of roots after wounding

Root formation after tissue injury is a type of plant regeneration known as *de novo* root regeneration (DNRR). DNRR from aboveground organs, such as hypocotyls, leaves and stems, are the fundamental of vegetative or clonal propagation, which is exploited in horticulture and forestry to produce large numbers of clones relatively quickly (Legué et al., 2014). When plants are injured, a series of responses are triggered to initiate injury repair and recovery, and JA serves as a key mediator in early signaling for DNRR (Zhou et al., 2019; Kim and Seo, 2025). JA rapidly accumulates after damage, thereby activating *ERF109*, an early JA-responsive gene, to upregulate the expression of *ASA1* to promote the production of auxin, which further drives AR initiation and outgrowth from leaf explants (Zhang et al., 2019; Liu et al., 2022). Moreover, JAZs physically interact with ERF109 to inhibit its activity to prevent hypersensitivity to wounding. Mutations of *ERF109* attenuated JA-induced LR formation and DNRR (Cai et al., 2014; Zhang et al., 2019), suggesting a central role of ERF109 in JA-mediated DNRR.

QC and stem cell niche re-establishment is involved in root regeneration; this process is also regulated by JA. JA promotes cell division of the QC through the RBR-SCR network and stress response protein ERF115. In addition, JA-induced *ERF109* transcription stimulates *CYCD6;1* expression, which participates in the activation of cell division, functions upstream of ERF115, and promotes regeneration of the QC and stem cell niche during root regeneration. Soil penetration and nematode herbivory induce JA-mediated wound responses and regeneration (Zhou et al., 2019). These studies elucidate the genetic network of JA in root tip repair via activation of the QC and stem cell niche.

## 4 Interaction of JA and other phytohormones in root growth

**Figure 2:**
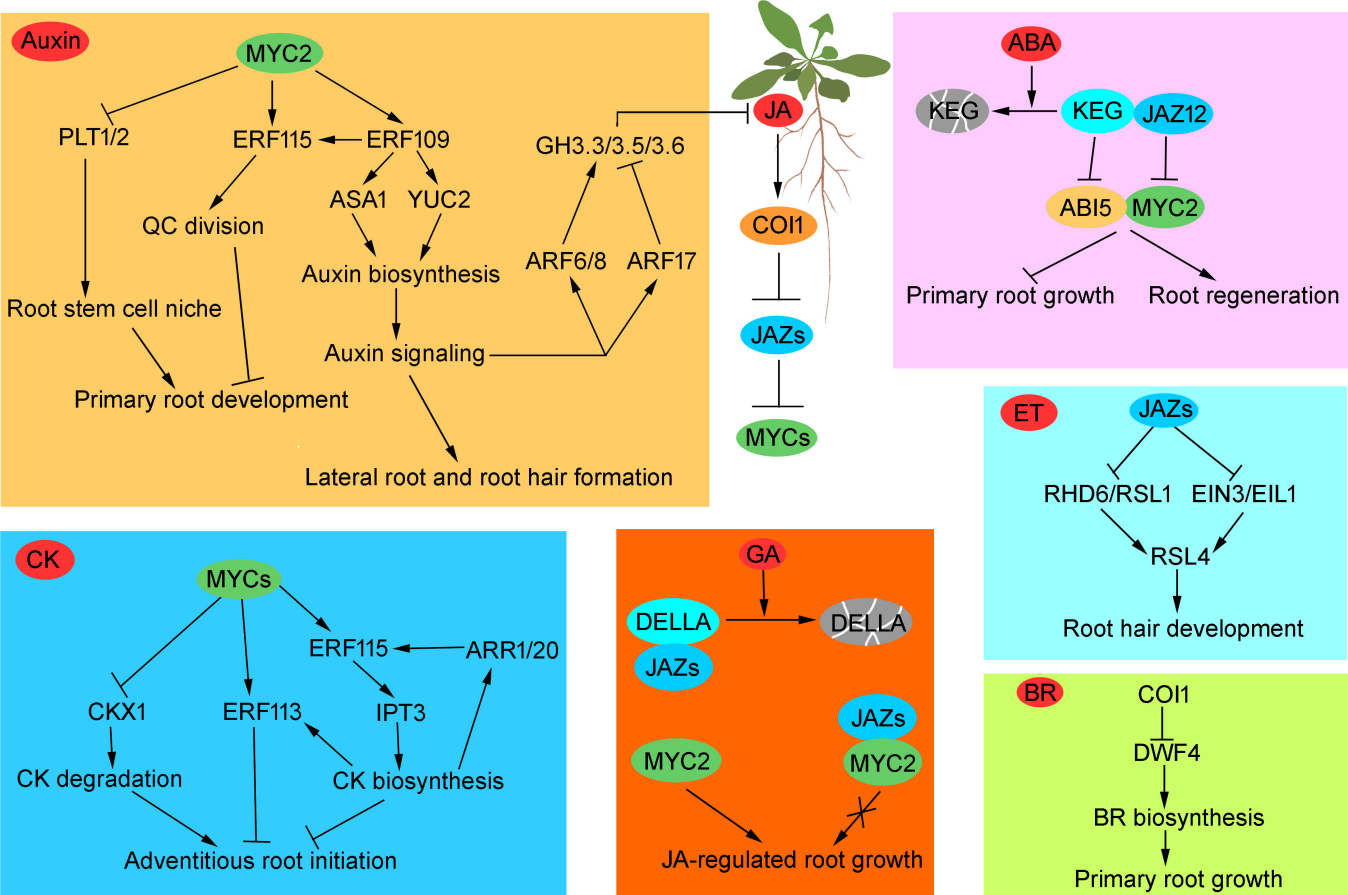
Integration of jasmonate (JA) and other phytohormones in *Arabidopsis* root development. JA inhibits primary root and adventitious roots growth, whereas promotes lateral roots and root hair development. JA exerts its regulatory roles on root growth by integrating with auxin, ethylene (ET), cytokinin (CK), abscisic acid (ABA), gibberellin (GA), and brassinosteroid (BR) biosynthesis or signaling pathways through its core signaling module COI1-JAZs-MYCs. Arrows denote positive regulation, whereas the T sharp symbol indicates inhibitory effects.

JA exerts its regulatory roles on root growth by integrating with auxin, ethylene, cytokinin, abscisic acid, gibberellin, and brassinosteroid biosynthesis or signaling pathways through its core signaling module (COI1-JAZ-MYC), at multiple levels including transcription, translation, and protein modification (**Figure 2**).

### 4.1 Auxin

Auxin governs every process in root development. Disruption of auxin biosynthesis, transport and signaling leads to abnormal root development (Qin and Huang, 2018). Multiple studies elucidated that JA employs auxin to steer root development (Cai et al., 2014; Sun et al., 2009). MeJA treatment induces the expression of genes related to auxin biosynthesis, transport, and signaling in roots, and thus participates in auxin-mediated primary root growth and LR initiation (Hentrich et al., 2013; Wang et al., 2009). Moreover, JA-induced LR formation was repressed in mutants with defective auxin biosynthesis and signaling (Sun et al., 2009; Xu et al., 2020), indicating that JA-mediated root development is auxin-dependent. At the molecular level, several transcription factors, such as ERF109, ERF115, PLT1/2, have been identified as downstream targets of MYC2, are serve as central regulators linking the crosstalk between JA and auxin (Cai et al., 2014; Chen et al., 2011; Sui et al., 2025; Zhou et al., 2019). Particularly, JA activates *MYC2* to repress the expression of *PLT1* and *PLT2*, which are key effectors for stem cell niche maintenance and root meristem activity and known to mediate developmental response to auxin in the root meristem, to inhibit primary root growth (Aida et al., 2004; Chen et al., 2011). ERF115, a rate-limiting factor of QC cell division, induces by JA and auxin in a synergistic manner to promoter root regeneration (Zhou et al., 2019). ERF109, acts upstream of ERF115 to promote regeneration, directly binds to the promoters of *ASA1* and *YUC2* to activate their expression, thus enhancing auxin biosynthesis to promote lateral root formation. Overexpression of *ERF109* produced much longer and more root hair. Knockout of *YUC2* partially alleviated the root hair phenotype caused by *ERF109* overexpression (Cai et al., 2014). In addition, auxin modulates JA homeostasis by regulating *Gretchen Hagen3* (*GH3*) genes, *GH3.3*, *GH3.5*, and *GH3.6*, through AUXIN RESPONSE FACTOR 6/8/17 (ARF6/8/17), then influences adventitious root formation (Gutierrez et al., 2012). Taken together, these studies suggest that there is a feedback regulation between JA and auxin, and the ERF109 regulatory module might be a key node in JA-auxin crosstalk in root development.

### 4.2 Ethylene

Ethylene as a gaseous plant hormone plays an important role in root growth and development. Generally, ethylene inhibits primary root elongation and LR formation, but promotes AR and RH growth (Li et al., 2024b; Qin and Huang, 2018; Qin et al., 2022), which is consistent or opposite to the role of JA in root growth (Han et al., 2023), implying that ethylene and JA antagonistically or synergistically to regulate root development in different root tissues and stages. ETHYLENE INSENSITIVE 3 (EIN3) and EIN3-LIKE1 (EIL1) are two master transcription factors mediating the ethylene signaling transduction, are also required for JA-inhibited root elongation (Zhu et al., 2011). The *ein3eil1* mutant was less sensitive, whereas transgenic plants overexpressing *EIN3* or *EIL1* were hypersensitive, to JA-induced inhibition of root elongation (Zhu et al., 2011). Further analysis of the associated mechanism revealed that JAZ proteins directly interact with EIN3/EIL1 and recruit an RPD3-type histone deacetylase (HDA6) as a corepressor to repress EIN3/EIL1-dependent transcription, and JA enhances the EIN3/EIL1 functions by removal of JAZ proteins (Zhu et al., 2011). This mechanism may also involve in regulating RH growth. Previous studies demonstrated that EIN3 interacts with RHD6 to co-activate *RSL4* expression to promote RH formation and elongation (Feng et al., 2017; Qiu et al., 2021), whereas JAZs interact with RHD6 and RSL1 to repress RH development (Han et al., 2020). Based on these studies, we speculate that JA promotes the degradation of JAZ proteins, thereby relieving the repression of JAZ proteins on the transcriptional activity of RHD6/RSL1 and EIN3/EIL1, which then coordinately regulate the expression of genes essential for RH growth.

### 4.3 Cytokinin

Cytokinins (CKs) are adenine-derived phytohormones that regulates cell division and differentiation in plants (Dello Ioio et al., 2007). Accumulating studies showed that JA and CK act synergistically in regulating root growth (Dob et al., 2021; Lakehal et al., 2020). Exogenous JA treatment inhibited the expression of *CYTOKININ OXIDASE/DEHYDROGENASE 1* (*CKX1*), which encodes an endoplasmic reticulum-localized enzyme involved in CK degradation, leading to the accumulation of CK to inhibit AR initiation, and the COI1-MYC2 module is required for JA-suppressed *CKX1* expression (Dob et al., 2021). Moreover, JA and CK synergistically activate the expression of the *ERF113*, a negative regulator of AR initiation, to inhibit AR initiation (Dob et al., 2021). ERF115, induced by JA and a direct target of MYC2, functions as a repressor of AR initiation by activating the expression of *ATP/ADP ISOPENTENYLTRANSFERASE 3* (*IPT3*) to control the *de novo* CK biosynthesis (Lakehal et al., 2020). Interestingly, the *ERF115* promoter contains a CK-responsive motif, and a yeast one-hybrid screen has shown that two type-B *Arabidopsis* response regulators (ARRs), ARR1 and ARR20, bind to the promoter of *ERF115* (Ikeuchi et al., 2018). The type B ARRs are transcription factors that act as positive regulators in the two-component CK signaling pathway (Argyros et al., 2008), suggesting that CK signaling may also control the abundance of *ERF115* transcripts, thus forming a feedback loop in AR initiation. Further exploring the mechanism underlying the synergism between JA and CK signaling will shed light on AR initiation regulatory processes.

### 4.4 Gibberellin

The interaction between JA and gibberellin (GA) in plant growth and development is primarily characterized by antagonistic effects (MaChado et al., 2017; Yang et al., 2012). Generally, GA promotes plant growth, whereas JA often inhibits growth, particularly under adverse stress conditions (Wang et al., 2021). At the molecular level, the antagonism between the JA and GA signaling pathways is primarily mediated by the interaction of JAZ proteins with DELLA proteins (Hou et al., 2010). DELLA proteins serve as negative regulators of the GA signaling pathway (Harberd, 2003; Silverstone et al., 2001). It interacts with JAZ proteins to release MYC2 from JAZ/MYC2 complex, thereby enhancing the ability of MYC2 to regulate its target genes. GA triggers degradation of DELLAs, which allows JAZ1 to bind MYC2 and attenuates MYC2-dependent JA signaling. Loss of function of DELLAs decreases the sensitivity of root to exogenous JA (Hou et al., 2010). Similar mechanisms also exist in rice. OsSLR1 is the only DELLA protein in rice, JA delays GA-mediated OsSLR1 protein degradation, and the *slr1* mutant is less sensitive to JA-induced growth inhibition (Yang et al., 2012). OsJAZ9 interacts with OsSLR1 to mediate the antagonistic interaction between JA and GA signaling. Knocking out OsJAZ9 weakened GA-promoted growth, whereas overexpression of *OsJAZ9* enhances the GA response (Um et al., 2018). Taken together, these studies provide a mechanistic understanding on how JA and GA signaling could be fine-tuned by each other through the interaction of JAZs and DELLAs.

### 4.5 Abscisic acid

Abscisic acid (ABA) is a well-known stress phytohormone which promotes adaption partly by modifying root growth (Belda-Palazón et al., 2022; Xiong et al., 2025). Several studies have shown that ABA interacts with JA to modulate root growth (Lackman et al., 2011; Pauwels et al., 2015). Transcriptome analysis ABA and JA responsive genes in rice shoot and root showed that half of the ABA-dependently expressed genes were also regulated by JA (Kim et al., 2018), suggesting that ABA and JA act synergistically in terms of gene expression regulation. PYRABACTIN RESISTANCE-LIKE4 (PYL4) and PYL5, two genes encoding ABA receptors, were induced by JA. Mutation of PYL4 and PYL5 causes altered JA responses (Lackman et al., 2011). MYC2, core transcription factor of JA signaling pathway, has been identified as a positive regulator of ABA signaling. Transgenic plants overexpressing *MYC2* exhibited higher sensitivity to ABA (Abe et al., 2003). Furthermore, MYC2 interacts with ABA signaling transcription factor ABA INSENSITIVE5 (ABI5) to modulate root regeneration (Wan et al., 2025). The RING-type ubiquitin E3 ligase KEEP ON GOING (KEG), which negatively regulates ABA signaling via its suppressive effects on the ABI5 accumulation, directly interacts with and partially inhibits the degradation of JAZ12 during JA-mediated root growth inhibition. ABA treatment promotes KEG self-ubiquitination and degradation, leading to an increase in ABI5 levels and a decrease in JAZ12 levels (Liu and Stone, 2010; Pauwels et al., 2015), suggesting that KEG is a key node in the integration of ABA and JA.

### 4.6 Brassinosteroid

Brassinosteroid (BR) is a family of polyhydroxylated steroid hormones involved in many aspects of plant growth and development (Planas-Riverola et al., 2019). Mutants with defective in BR biosynthesis or signaling exhibited retarded root growth (Planas-Riverola et al., 2019; Vukasinovic et al., 2021). The relationship between BR and JA is not fixed; it shifts between antagonistic and synergistic depending on the developmental process or environmental condition. Under normal conditions, BR signaling can attenuate the JA-induced inhibition of root growth, creating a balance that prevents defense responses from completely stalling development (Gan et al., 2025); when exposed to toxic heavy metals like Arsenic (As), both BR and JA could work together to mitigate damage. They help re-establish root growth and form barriers. For instance, xylogenesis was promoted by MeJA as a mechanical defense barrier against arsenate, while Epibrassinolide (eBL) counteracts the negative impact of arsenite on root formation (Della Rovere et al., 2025). Through screening for mutants that could suppress *coi1* insensitivity to JA-inhibited root growth, *partially suppressing coi1* (*psc1*) mutant was isolated (Ren et al., 2009). *PSC1* is an allele of *DWARF4* (*DWF4*), which encodes a key enzyme in BR biosynthesis. Mutation in *DWF4* leads to JA hypersensitivity. Moreover, JA treatment inhibited the expression of *DWF4*, and this inhibition was dependent on COI1. Exogenous BR treatment can attenuate the inhibitory effects of JA on root growth (Huang et al., 2010; Ren et al., 2009). These studies suggest that BR acts downstream of JA to regulate root growth, which extends our understandings on the JA signal transduction.

## 5 Conclusions and perspectives

The high developmental plasticity of root not only enabling plants to forage available water and nutrients from the heterogenous soil, but also improving plant survival under various dynamic environmental conditions (Karlova et al., 2021). Therefore, dissecting the regulatory mechanism of root growth can contribute to produce stress-tolerant crops with stable yields even in challenging environments. Numerous studies showed that JA plays an important role in root development, and it exerts this function through interacting with other phytohormones (Han et al., 2023) (**Figure 2**). However, the complete network of JA and other phytohormones orchestrating root development is far from elucidated. Future studies should dissect the precise mechanisms of their interactions in a multidimensional space by employing single-cell sequencing, spatial transcriptomics, real-time imaging, and synthetic biology approaches, combined with AI-driven prediction, which will greatly promote research on hormone crosstalk, and provide potential targets for the cultivation of crop varieties with smart root and superior stress resistance.

JA is a critical phytohormone that regulates various aspects of plant development and stress responses (Huang et al., 2017; Wasternack and Hause, 2013). An appropriate level of JA is essential for its biological functions, while excessive JA accumulation would trigger an over-activation of the defense machinery, which accompanied by a reduction in growth and reproductive outputs (Li et al., 2022). This growth-defense tradeoff mechanism allows plants to reallocate limited resources based on environmental stresses, thereby optimizing their fitness (He et al., 2022). How to uncouple the growth-defense tradeoff is crucial for cultivation crops that combine robust defenses while maintaining normal yields. Recent studies have shown that modification of key components in JA signaling pathway uncoupled the growth-defense tradeoff (Li et al., 2024a; Xiao et al., 2025), opening broad avenues to obtain cultivars with enhanced yield without compromised defenses. Further identification of the favorable alleles of JA biosynthesis and signaling components would offer potential targets for marker-assisted selection and genome editing in future crop improvement.

In the past decades, plant breeders have already made huge gains by manipulating above ground traits, but the same is not true for root traits, as roots are encased in soil and cannot be visualized, digging them up is a time-consuming and sometimes back-breaking process, and the root phenotype is more prone to vary in different growth conditions compared with the aboveground traits. Until now, studies on root growth are mainly carried out in seedlings grown in hydroponic and gel/agar systems, which is distinct from roots grown in real soil conditions. With the development of technology, applying new approaches such as X-ray computed tomography (CT) imaging to study the root system architecture in natural soils and in complex environments would enable more reliable measurement of root traits and the identification of related genes. However, CT requires specific equipment and a high expense for image collection. The development of simple, fast, and low-cost detection methods will greatly accelerate the research of root development.
